# Effects of Particle Size on Mechanical Properties and Forming Accuracy of *Prosopis chilensis* Powder/Polyethersulfone Composites Produced via Selective Laser Sintering

**DOI:** 10.3390/polym16131786

**Published:** 2024-06-25

**Authors:** Alaaeldin A. A. Abdelmagid, Aboubaker I. B. Idriss, Chun-Mei Yang

**Affiliations:** 1 School of Civil Engineering, Quanzhou University of Information Engineering, Quanzhou 362008, China; alaaeldin@qzuie.edu.cn; 2 College of Mechanical and Electrical Engineering, Northeast Forestry University, Harbin 150040, China; ycmnefu@126.com; 3 Department of Mechanical Engineering, Faculty of Engineering Science, University of Nyala, P.O. Box 155, Nyala 63312, Sudan

**Keywords:** composite material, polyethersulfone, *Prosopis chilensis*, particle size, selective laser sintering

## Abstract

Wood–plastic composites are becoming increasingly recognized for their sustainability and their potential for use in various production processes. Nevertheless, enhancing their mechanical strength continues to be a difficult challenge. The objective of this research was to improve the mechanical strength of wood–plastic composite components manufactured through selective laser sintering (SLS). This was achieved by integrating a sustainable composite material, *Prosopis chilensis* (PCP), with polyethersulfone (PES) to form a composite referred to as PCPC. This study showcased the effect of various PCP particle sizes on mechanical strengths, dimensional accuracies (DAs), and surface roughness of PCPC parts manufactured using AFS-360 SLS. Single-layer sintering was employed to assess PCPC powder’s formability with varying PCP particle sizes, and various tests were conducted to understand the materials’ thermal properties and analyze particle dispersion and microstructure. The results demonstrated that PCP particle sizes ≤ 0.125 mm significantly enhanced the mechanical strength, forming quality, and DA compared to other particle sizes and pure PES. Key findings for PCPC parts with PCP ≤ 0.125 mm included a bending strength of 10.78 MPa, a tensile strength of 4.94 MPa, an impact strength of 0.91 kJ/m^2^, and a density of 1.003 g/cm^3^. Post-processing further improved these parameters, confirming that optimizing PCP particle size is crucial for enhancing the mechanical properties and overall quality of PCPC parts produced via SLS.

## 1. Introduction

In 1988, Deckard, affiliated with the prestigious University of Texas at Austin, introduced selective laser sintering (SLS), an innovative additive manufacturing (AM) process [1,2]. SLS is a computer-aided manufacturing (CAM) technique utilized in AM to construct 3D components based on a computer-aided design (CAD) model [3]. Thus, through the use of SLS technology, parts with complex designs and shapes can be manufactured, resulting in high-accuracy components [4,5,6]. SLS offers a number of benefits over conventional industrial methodologies: (1) SLS technology possesses the capability to produce intricate and elaborate shapes and designs that are unattainable through conventional manufacturing methods; (2) the SLS method demonstrates the ability to transfer rapidly from design to fabrication (from CAD to CAM), often within hours; (3) SLS technology operates without the requirement for support structures during the manufacturing process, thereby enhancing efficiency and streamlining production procedures [7,8]. These three characteristics of SLS technology contribute to its attractiveness as a prominent manufacturing method [9]. Manufacturers and companies continuously explore novel materials in materials science and technology to meet consumer demands and societal needs for various products, particularly in manufacturing applications [10]. At present, the primary focus of SLS materials lies in polymers, ceramics, metals, and their respective composites [11,12,13,14,15,16,17,18]. Nonetheless, the high cost of material preparation technology hampers the widespread application of SLS. Therefore, there is an imperative demand to develop new environmentally friendly materials that are natural, energy-efficient, and cost-effective [19,20]. The application of the SLS technique is applicable to any materials that exist in powder form or have the ability to be melted and fused [14,21,22]. Thus, in recent times, SLS technology has found application in manufacturing a wide range of products, including medical equipment, construction components, industrial goods, automobiles, and wood-based materials [23,24].

Currently available SLS parts have limited and often low-quality applications [25]. Therefore, it is crucial to establish the appropriate particle size range of raw materials, optimal mixture ratio, and SLS processing parameters to guarantee the excellence of the fabricated sintered components. Accordingly, the excellence of SLS parts is contingent on both the laser beam and biomass composite powder interaction, as well as the powder particle size and SLS processing parameters [26,27]. Consequently, conducting a thorough investigation to determine the ideal powder particle size’s influence on the dimensional accuracy (DA), surface quality, and mechanical strength of SLS-sintered parts becomes essential.

Several researchers have conducted studies on the SLS processing of biomass powder composites [14,28,29]. Guo et al. [30] from Northeast Forestry University (NEFU) proposed a new environmentally friendly method that involves using cost-effective natural agricultural and forestry waste as a feedstock for SLS. This innovative approach addresses the issues of limited raw material availability and high costs, while also contributing to the reduction of pollution from waste disposal and CO_2_ emissions. Prior research has primarily concentrated on expanding the applications of existing SLS materials [31]. However, there is still a lack of a comprehensive understanding of how varying raw material particle sizes impact particle spreading and mechanical characteristics (comprising surface roughness, DA, and strength). 

*Prosopis chilensis* (PCP), a renewable biomass material derived from forestry waste, offers numerous advantages, including lower cost, reduced carbon emissions, and higher mechanical strengths compared to other wood powder composites like rice husk and sisal fiber composites [5,14]. Due to these benefits, green PCP was selected as a raw component for the polyethersulfone (PES) matrix in the SLS process. Additionally, PCP trees are abundant in various regions of Sudan, particularly in the northern, eastern, and central areas, and their uncontrolled growth has led to the destruction of agricultural projects, rendering several agricultural areas unsuitable for farming. To tackle this challenge, the government in these regions has resorted to mass burning of *Prosopis chilensis* trees, causing ecological degradation. PES, also known as a thermoplastic polymer, is gaining prominence as a highly promising substitute for thermoplastic-based polymers in various engineering applications [31].

Previous research on PCP/PES composite (PCPC) SLS parts has shown favorable properties [32]. However, technical defects persist during the PCPC SLS processing, including deformations on the upper surface of sintered parts and compromised mechanical strength and DA of the resulting SLS parts. These defects are attributed to the utilization of unsuitable particle sizes. As a result, the primary objective of this paper was to investigate the influence of PCP particle size on the spreading, DA, impact strengths, tensile strengths, and bending strengths of PCPC fabricated through SLS.

In this study, a range of PCP particle sizes were examined, specifically ≤0.149 mm, ≤0.125 mm, ≤0.105 mm, and ≤0.088 mm. The chosen mixture ratio for the investigation was 10/88 (wt/wt). Additionally, to enhance the surface quality and mechanical strengths of the PCPC, post-processing infiltration utilizing wax was performed. The mechanical characteristics of the PCPC SLS parts were subjected to a comparative analysis with earlier published findings to validate the experimental findings. Moreover, a comparison between the actual PCPC part and the simulated counterpart was carried out to verify the testing process. 

## 2. Materials and Methods

### 2.1. Raw Materials

**Figure 1:**
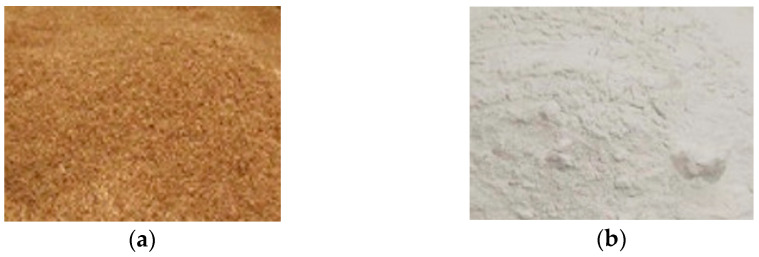
Raw materials of (**a**) PCP and (**b**) PES.

The present study employed PES and PCP in powder form as the raw materials. The raw *Prosopis chilensis* sample was sourced from the White Nile State of Sudan. The *Prosopis chilensis* was processed into PCP utilizing crushing machinery (Jiangsu Guibao Group Co., Ltd., Beijing, China). The PCP particles were carefully sieved through multiple mesh screens, including 80, 100, 120, and 140 mesh screens, using a vibrating sifter (Model ZS 350 Jiangsu Guibao Group Co., Ltd., Beijing, China) to eliminate agglomerated particles. An intensive shaking procedure using a standard vibrating sifter was employed to eliminate agglomerated particles, yielding PCP particles of various sizes. The PES powder was sourced from a company in Shanghai. PES, a thermoplastic polymer, exhibits several key features as a powder material, including an irregular block particle shape, a particle diameter ranging from 0 to 58 µm, and a density ranging from 0.7 to 1.32 g/cm^3^. After the sieving process, the PCP underwent drying at 100 °C using electrical heating for a duration of 4 h. Subsequently, the dried PCP was manually combined with PES powder, and a high-speed mixer was used to achieve a high level of uniformity and obtain homogeneous powder mixtures with maximum dispersion and consistent color. The PCPC powder was mixed in a high-speed mixer for 15 min, which is sufficient to achieve full homogeneity, and the speed of high-speed mixer ratio is 1000 to 3000 RPM (revolutions per minute). Figure 1a illustrates the pure PCP powder, while Figure 1b displays the pure PES powder.

### 2.2. Preparation and Characterization of PCPC

**Figure 2:**
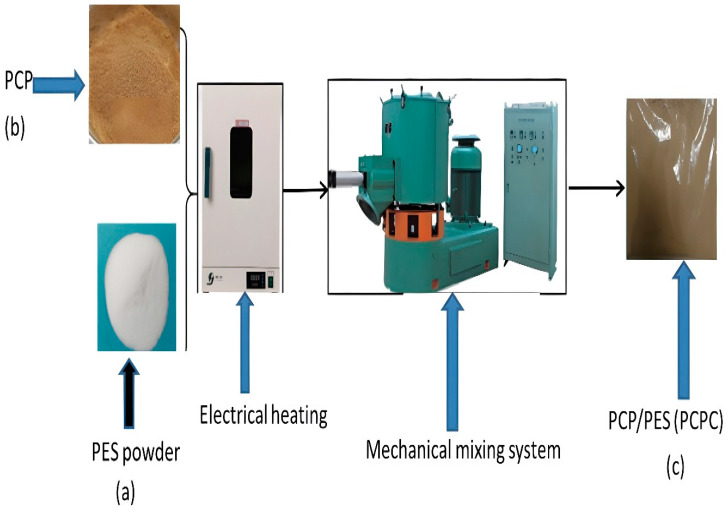
Preparation process of PCP/PES (PCPC) composite powder: (**a**) pure PCP, (**b**) pure PES, and (**c**) the composite of PCPC.

After manually combining the dried powders of PES and PCP in PCPC, the mixture underwent mechanical mixing in a high-speed mixer which ranges from 1000 to 3000 RPM for 15 min. PCPC with PCP/PES ratios of 10/90% across four different PCP particle sizes was prepared for SLS testing. Following mixing, the prepared PCPC was placed in an oven for one hour to remove moisture. PCP tended to enhance the PCPC’s surface smoothness, flexibility, accuracy, and mechanical strength. Figure 2 illustrates the procedural flow chart for preparing the PCPC composite powder.

### 2.3. SLS Experiment

**Figure 3:**
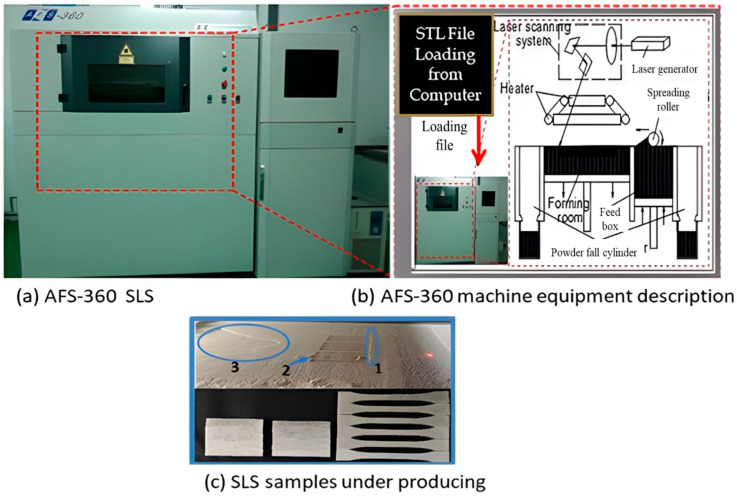
The equipment and description of the components that integrate with the printer AFS-360 during product printing. In (**c**) 1 = Finishing and smoothing the powder in the working area in order to start the laser moulding process; 2 = Initiating the laser moulding process above the surface of the powder; 3 = Covering the formed shape with the laser with a layer of fine powder until the final shape is complete.

The SLS experiments were carried out on an AFS-360 selective laser sintering (SLS) machine, manufactured by Beijing Longyuan Technology Ltd., Beijing, China, to sinter the PCPC parts with various PCP particle sizes. Figure 3 illustrates the equipment and its components used in the AFS-360 printer during the product printing process: (a) the ASF-360 SLS machine, (b) the description of the SLS machine, and (c) the sample powder under printing. The fundamental process of the SLS experiment involves heating a flat surface of PCPC powder to a temperature just below or near its melting point. Subsequently, the particles forming the desired cross-section are melted using a laser. The surface is then lowered, and a fresh layer of powder is applied over the previous one, initiating the process once more, as depicted in Figure 3c. This sequence is iterated numerous times, often reaching thousands of repetitions, to construct a complete part.

The design of STL-formatted tensile specimens followed the guidelines outlined in the GB/T1040-1992 standard (*Plastics-Determination of tensile properties*. China Oil and Chemical Industry Association: Beijing, China, 1992), with dimensions measuring 0.15 m × 0.02 m × 0.01 m. Similarly, the STL format for bending specimens adhered to the specifications of the GB/T9341-2008 standard(*Plastics—Determination of flexural properties*. AQSIQ; SAC: Beijing, China, 2008), featuring dimensions of 0.08 m × 0.013 m × 0.004 m. Additionally, the STL representation of impact specimens was established in accordance with the ISO179-2000 standard [33], with dimensions measuring 0.08 m × 0.01 m × 0.004 m. The fabrication of specimens comprising both pure PES and PCPC composites was conducted using an AFS-360 apparatus, which was outfitted with a 55 W carbon dioxide laser. The laser had a wavelength of 0.0106 m and a laser beam diameter of 2.6 ± 0.4 mm.

### 2.4. Mechanical Testing

**Figure 4:**
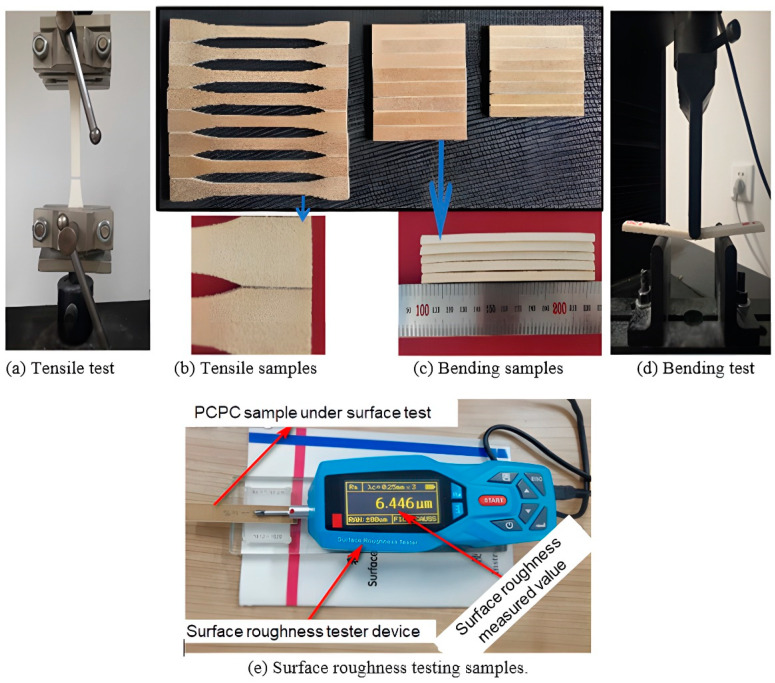
The testing of mechanical properties and the testing samples.

Bending and tensile mechanical testing were conducted using a Byes-3003 universal testing machine obtained from TMS Company, Shenzhen, China. The impact strength was evaluated in accordance with ISO179-2000. An impact sample with dimensions of 0.08 m × 0.01 m × 0.004 m was used for the testing. The pendulum impact energy applied during the test was 2 J, with a span length of 0.064 m. Additionally, the velocity of the impact pendulum was measured to be 2.9 ${\mathrm{ms}}^{-1}$. The tensile strength sample dimensions were 0.15 m × 0.02 m × 0.01 m, and the testing was conducted following the GB/T1040-1992 standard. The crosshead speed during the test was set at 0.005 m/1 min, and a gauge length of 0.05 m was employed. The bending strength samples were of dimensions 0.08 m × 0.013 m × 0.004 m and were subjected to testing following GB/T9341-2008. A span length of 0.08 m was used, and the tests were run at a crosshead speed of 0.0001 m/1 min. Figure 4 showed the testing of mechanical properties and the testing samples, i.e., (a) tensile sample under testing, (b) tensile sample, (c) bending sample, (d) bending sample under testing, and (e) surface roughness sample under testing. The impact strength, bending strength, and tensile strength tests were performed three times each, and a 95% confidence interval was generated for the statistical analysis of the average data.

Prior to conducting the SLS experiments with PCPC, a preliminary single-layer sintering test was employed to validate the suitability of employing the PCPC composite powder within the SLS manufacturing process. Through a lot of single-layer experiments of four different PCPC parts and pure PES, the evaluation of suitable particle sizes for the PCP particles was performed under consistent mixture ratios and processing parameters. Additionally, mechanical assessments were employed to determine the optimal particle size for the powder. The most favorable sintering outcomes were attained when utilizing PCP particles with a size of ≤0.125 mm, exhibiting superior performance in comparison to pure PES powder. The most effective parameters for the SLS procedure were determined to be as follows: a processing temperature maintained at 75 °C, a preheating temperature set at 80 °C, a scan spacing of 0.16 mm, a layer thickness measuring 0.14 mm, a scanning speed of 1800 mm/s, and a laser power setting of 12 W.

### 2.5. Dimensional Accuracies (DAs)

The dimensional measurements of 0.08 m × 0.013 m × 0.004 m were employed for conducting a DA analysis on various PCPC parts. This DA analysis facilitates the determination of the ideal particle size range of the PCP concerning both mechanical strengths and DA. The DAs of the PCPC SLS were determined through Equation (1). The measurements for the actual sizes, namely the length (x), width (y), and thickness (z), were obtained using a Vernier caliper manufactured in China, known as the Industrial Grade IP67 Waterproof Digital Vernier Caliper.

(1) $$\delta \left(\%\right)=\left(1-\frac{\left[\mathrm{Lo}-L\right]}{\mathrm{Lo}}\right)$$

Lo represents the specified dimension; L denotes the measured dimension; δ (%) represents the DA expressed as a percentage.

### 2.6. Density

Density analyses were conducted on bending specimens with specific measured dimensions of 0.08 m × 0.013 m × 0.004 m to determine the PCPC parts’ density with various PCP particle sizes. The density calculations were based on the weights and dimensions of the PCPC parts, which were determined using an electronic balance and a Vernier caliper. Equation (2) is used to calculate the densities of PCPC SLS parts.

(2) $$\rho =\frac{w}{l.b.h}$$

w stands for the mass of the SLS part in grams; h signifies the thickness in mm; b is the width in mm; l denotes the length in millimeters; and ρ represents the density in g/cm^3^.

### 2.7. Microstructure Characterization

**Figure 5:**
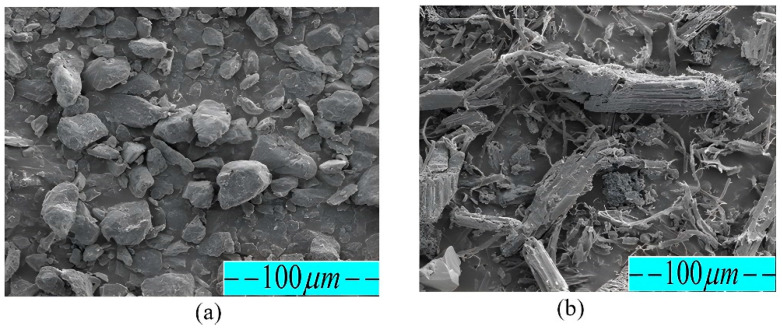
SEM images of (**a**) pure PES and (**b**) pure PCP.

Scanning electron microscopy (SEM) analysis was performed to scrutinize the cross-sectional morphology of pure PCP and PES samples. The morphology was examined using the FEI Quanta 200 Scanning Electron Microscope (SEM) from Hewlett-Packard Company in Amsterdam, Netherlands. The specimen was mounted onto conductive adhesive tape and subjected to a gold-spraying process. Subsequently, the prepared sample was introduced into the SEM to facilitate the observation of surface morphology. The results are visualized in Figure 5.

### 2.8. Thermogravimetric Analyzer (TGA) Test of PES and PCP Powders

**Figure 6:**
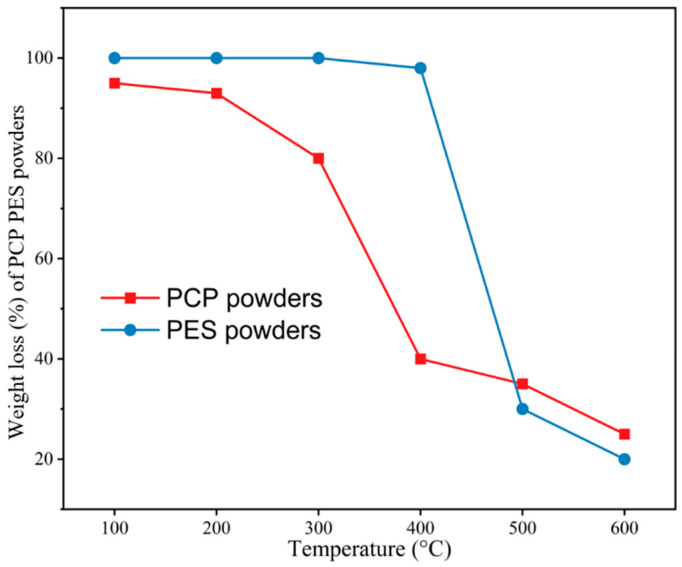
TGA curve of PES and PCP powders.

The Pyris 6 thermogravimetric analyzer (TGA) manufactured by PerkinElmer was employed for the examination of PES and PCP. The TGA test was performed using specific parameters: 10 mg sample masses of PES and PCP underwent thermogravimetric analysis at a heating rate of 10 °C/min. A temperature range of 100 to 600 °C was covered by this test. Figure 6 illustrates the TGA curve delineating the behavior of PES and PCP.

### 2.9. Differential Scanning Calorimetry (DSC)

**Figure 7:**
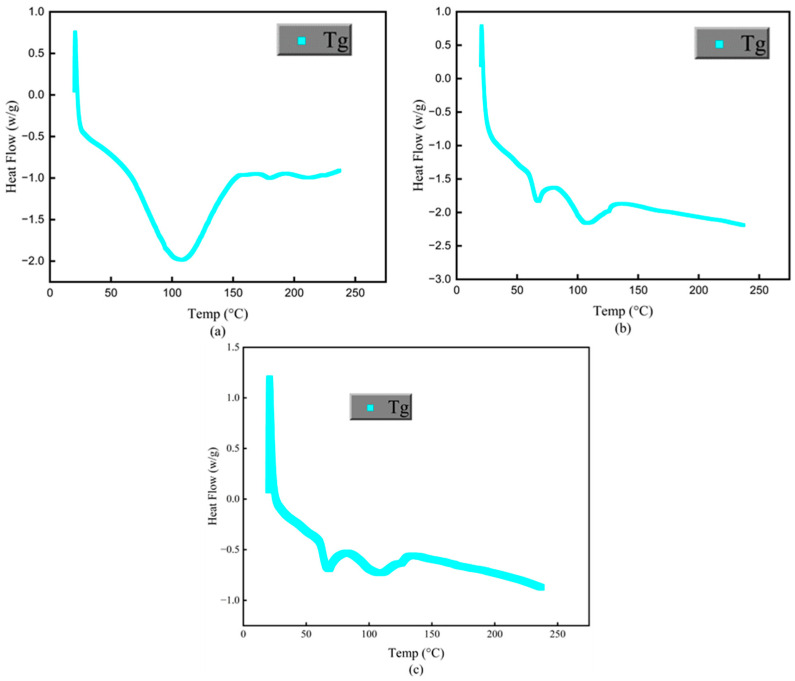
DSC curves (Tg) of (**a**) PCP, (**b**) PES, and (**c**) PCPC.

The DSC technique was employed using a PerkinElmer instrument from Waltham, MA, USA, to ascertain the glass transition temperatures (Tg) of both PCP and PES powders. The combined mass of both powders fell within the range of 3–5 mg. A temperature range of 100 to 600 °C was covered by this test. The DSC curves representing the behavior of the powders are presented in Figure 7.

### 2.10. Post-Processing

**Figure 8:**
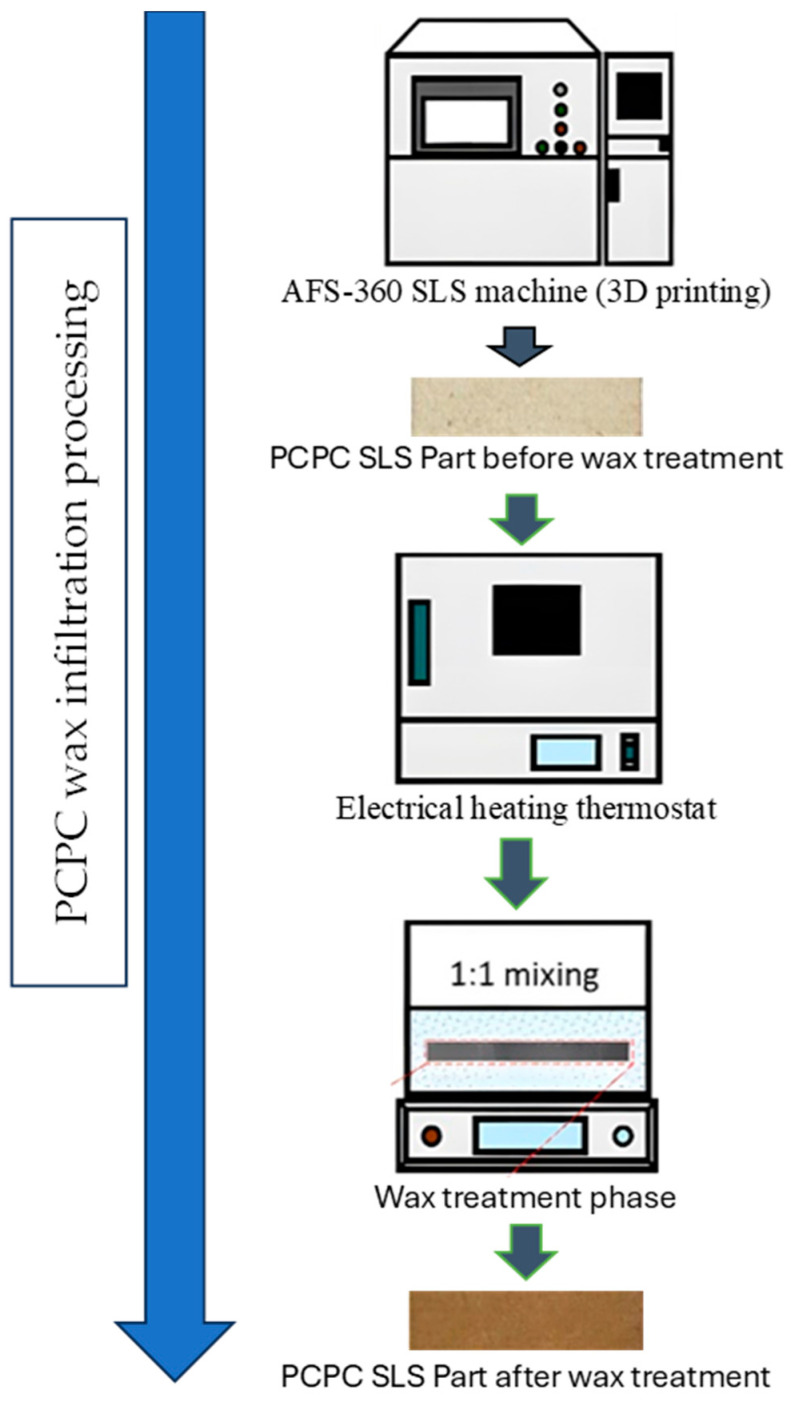
PCPC wax infiltration.

For the purpose of enhancing both the upper-surface quality and mechanical strength of PCPC SLS parts, a post-processing technique, specifically wax infiltration, was employed. The procedural steps for this post-processing approach are delineated in Figure 8. Different PCPC parts were extracted out of the AFS-360 SLS, subjected to meticulous cleaning, and subsequently positioned within a thermostat-controlled electric heater to be insulated at a temperature of 70 °C. After the insulating phase, the designated PCPC parts were gradually submerged in a wax pool, facilitating the penetration of wax into the pores through the process of capillarity. As the wax fills the interstitial gaps between particles, it forms a strong bond with the PCPC particles, resulting in increased mechanical strength and density, along with enhanced surface quality. Following the wax infiltration, the dimensions of the PCPC SLS parts experienced only marginal expansion in all directions. This observation confirms that the post-processing exerted minimal influence on the DA of the sintered PCPC parts. To mitigate this concern, a polishing procedure is introduced to enhance the DA of the wax-treated PCPC SLS parts.

### 2.11. Surface Roughness Test

The surface roughness measurement was conducted on the upper surface of the PCPC sintered parts, which possessed dimensions of 0.08 m × 0.013 m × 0.004 m. This analysis was performed utilizing a surface roughness tester and encompassed various PCP particle sizes.

## 3. Results and Discussion

### 3.1. Thermal Performance Analysis of the PCPC

Preheating powder material within a defined temperature range, as determined through DSC analysis, is crucial in mitigating warping, deformation, and compaction of PCPC sintered components during the SLS process. Thus, before laser sintering processing of PCPC powder, the laser sintering window of the PCPC composite could be determined through a DSC test. The operational dynamics of SLS technology are predominantly governed by thermal factors. Nevertheless, the absence of a distinct melting point in PCP leads to the significant involvement of the amorphous PES polymer powder in the creation process of PCPC components. Utilizing DSC analysis, the Tg of PES and PCP was established, which therefore provided valuable information for estimating the temperatures at which the powder has to be preheated. For quality assurance of fabricated PCPC SLS components, preheating of PCPC was conducted at a temperature derived from the DSC curve (Figure 7). The temperature range spanning from the softening point (Ts) to the caking temperature (Tc) is referred to as the sintering window. PES represents a non-crystallizable polymer, and Ts denotes the Tg for both types of powders. Regrettably, Tc cannot be ascertained via DSC analysis in this investigation; instead, it was directly observed during experimentation. Therefore, prior to conducting the DSC examination, it is imperative to establish the temperature of thermal decomposition for PCPC constituents. This necessitates the utilization of TGA, the outcomes of which are presented in Figure 6. The TGA analysis revealed initial decomposition temperatures of 298 °C for PCP and 408 °C for PES powder. Consequently, the DSC testing temperature for varying PCP particle sizes was set below 298 °C. Therefore, the experimental DSC temperature varied between 20 and 240 °C, remaining below 298 °C. As indicated in Figure 7, the Tg (Ts) value for PES was determined to be 88 °C, while for PCP, it was established to be 89 °C. Consequently, the preheating temperature for PCPC must not exceed these respective Tg values. Experimental observations indicated full caking at 114 °C and 116 °C for PES and PCP samples, respectively. Accordingly, the PCPC preheating temperature was chosen as 80 °C, remaining below 88 and 89 °C. 

### 3.2. The Effect of PCP Particle Size on Spreading of the PCPC

**Figure 9:**
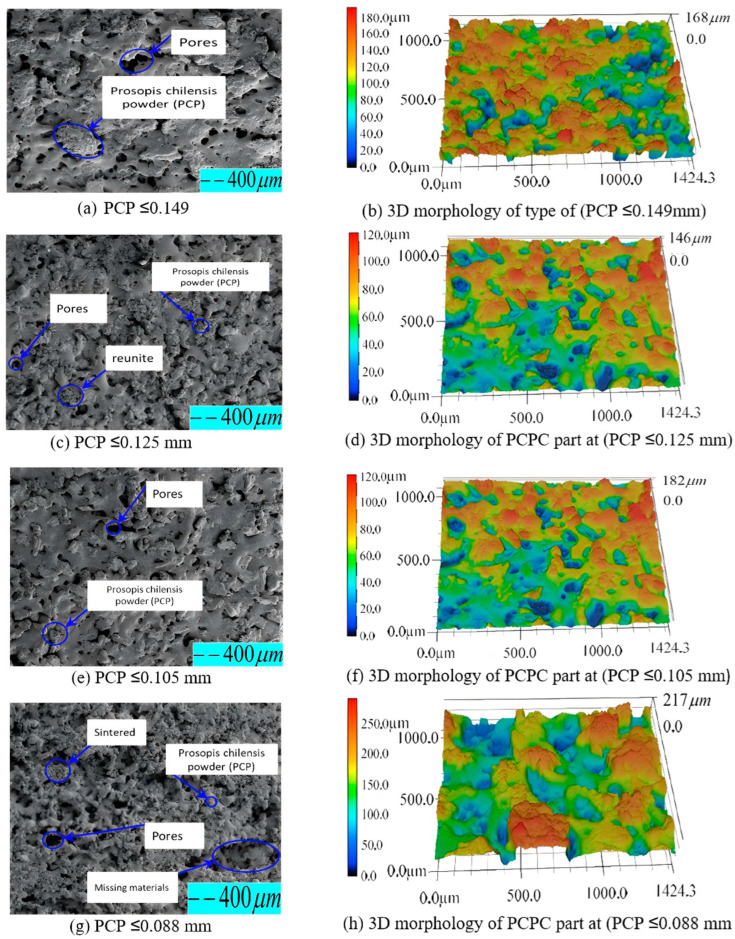
Images of the upper-surface morphology and three-dimensional representations of PCPC parts.

**Figure 10:**
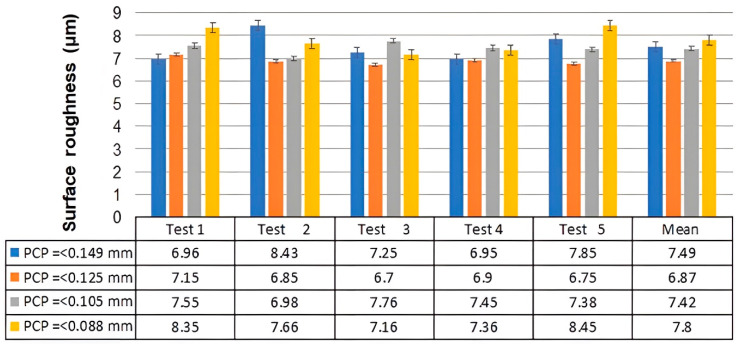
The PCPC surface roughness measurements.

Within the SLS process, biomass powder particle size and its spreading on the surface of the molded part are very important factors influencing sintered part surface quality. Consequently, the surface quality of the SLS parts directly impacts the performance and potential applications of the sintered composite powders. Figure 9 presents the surface and 3D morphology of parts fabricated with varying PCP particle sizes within PCPC. Correspondingly, Figure 10 illustrates the recorded top surface roughness measurements. Initially, a decrease in PCP particle sizes led to an improvement in surface quality of the sintered parts, where an ideal level of 6.87 µm was achieved at PCP ≤ 0.125 mm. Meanwhile, the upper surface showed a good surface with small holes and minimal powder particle agglomeration. Furthermore, there were no areas of missing materials on the upper surface of the PCPC molded part (Figure 9c,d). Thereafter, the surface quality decreased. The ideal PCP particle size improved the top surface sintering quality, inducing comprehensive PES blending and melting. This heightened the cohesion between PES powder and PCP, thereby enhancing the top surface and mechanical strength in sintered PCPC parts.

Figure 9a–d display the SEM images and three-dimensional morphology analysis depictions of the upper surface of the PCPC samples. The SEM images in Figure 9c,d show relatively small holes, and there is no missing materials area. When compared with Figure 9a,b, the distribution of PCP particles on the upper surface of the sintered part in Figure 9c,d is more even (Figure 9d). Due to an optimal particle size, there is no missing materials area in the PCPP sample; thus, the PCP particles are not prominent, and consequently, the upper surface of the part in Figure 9c,d is flat (Figure 9d), and the surface roughness value is the best one (Figure 10).

The PCP particle spreading on the upper surface of the molded part in Figure 9a is prominent, and the roughness is relatively large compared to Figure 9c. Therefore, PCPC parts with PCP ≤ 0.125 have fewer holes on the upper surface of the sintered parts, no agglomeration of PCP particles, and there are no materials missing, and the particles are evenly spreading.

Figure 9e,f (PCP ≤ 0.105 mm) also show many holes and agglomeration of PCP particles on the upper surface of the molded part; however, the missing materials area gradually decreases. Due to a small area of missing material, the upper surface of the PCPC molded part is still rough, but the value of roughness is smaller than PCP ≤ 0.088 mm, as displayed in Figure 10. Because of an increase in PCP particle size, the surface area of the particles decreases, and the adhesion between the particles is reduced, but there are still some PCP particles that agglomerate and adhere to the spreading roller. Consequently, the sintered part contains many pores, there is agglomeration of PCP particles, and a small area of material is missing in the molding of PCP ≤ 0.105 mm. (Figure 9e).

Figure 9g,h show that there are several holes, powder particle agglomeration, and large areas of missing materials on the upper surface of the PCPC molded part (PCP ≤ 0.088 mm). Due to a large area of missing material, the upper surface of the molded part becomes very rough, as exhibited in Figure 9g, and the surface roughness values are displayed in Figure 10. The main reason is that the PCP particles in PCP ≤ 0.088 mm are very small, which caused a large surface area of missing material due to a large adhesion between the particles (PCP and PES). Thus, the powder particles are easily agglomerated and adhere to the powder spreading roller. Therefore, there are many holes, agglomeration of PCP particles, and large areas of missing materials in the molded parts in Figure 9g.

Generally, in a very large and small PCP particle size, molten PES particles cannot entirely occupy inter-powder pores due to powder adhesion, resulting in larger holes on the upper sintered surface. The cumulative findings establish PCP less than or equal to 0.125 mm as the optimum particle size attributed to its uniform dispersion.

### 3.3. The Micromorphology Analysis of Various PCPC Samples

**Figure 11:**
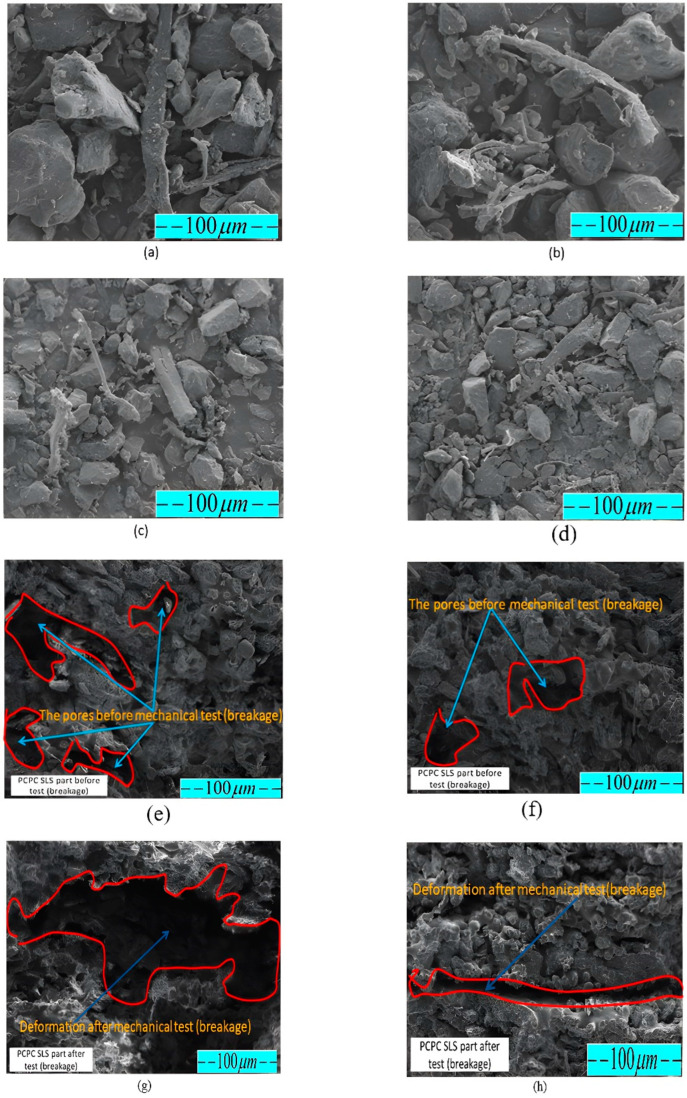
Surface morphology of (**a**) PCPC pieces with PCP ≤ 0.149 mm, (**b**) PCPC pieces with PCP ≤ 0.125 mm, (**c**) PCPC pieces with PCP ≤ 0.105 mm, and (**d**) PCPC pieces with PCP ≤ 0.088 mm. (**e**,**f**) SEM images of PCPC SLS parts before mechanical testing (PCP ≤ 0.149 and ≤ 0.125 mm, respectively); (**g**,**h**) shows the deformations of PCPC SLS parts after mechanical testing at (PCP ≤ 0.149 and ≤0.125 mm, respectively).

Powder particle dispersion and PCP-amorphous PES matrix interfacial binding significantly influence mechanical characteristics and potential application of PCPC parts. Therefore, an examination of particle size’s impact on internal microstructure of fabricated PCPC sintered parts is essential, along with identifying suitable PCP powder particle sizes. SEM images of PCPC samples with varying PCP particle sizes (PCP ≤ 0.149, ≤0.125, ≤0.105, and ≤0.088 mm), along with pure PES and PCP, are shown in Figure 5 and Figure 11. As shown in Section 3.4, the surface quality of SLS parts relies on powder material particle sizes. Thus, PCP particle size significantly impacts PCPC sintered parts’ microscopic morphology, surface quality, and mechanical strength. Pure PES powder exhibited a smoother, flatter surface, as displayed in Figure 5a, whereas pure PCP displayed an uneven, rough surface with non-uniform particle size, as illustrated in Figure 5b. The primary input for combining PCP and PES powders is laser power, and the particle size of PCP affects the power absorption of the PCPC composite. Thus, it is essential to determine the suitable particle size range of the PCP. PCPC SLS samples with PCP particle sizes of ≤0.149 and ≤0.125 mm pre- and post-mechanical testing were examined, which are exhibited in Figure 11e,f and Figure 11g,h, respectively. Figure 9a,b and Figure 11a show that most of the PCP is covered by the PES matrix, and the interface bonding is relatively good. The numbers of internal pores in the PCPC sintered part are relatively large, and the density is relatively low.

Figure 9c,d and Figure 11b reveal a uniform PCP distribution in the PES matrix without aggregation or surface material gaps in sintered parts. Furthermore, small internal pore quantities and sizes, substantial sintering necks between PCP and PES, and continuous sintering zones enhance mechanical strength alongside density of the sintered PCPC part (PCP ≤ 0.125 mm), as shown in Figure 11a–d. However, the upper surface of PCPC parts manufactured using PCP ≤ 0.149 mm exhibits larger internal pores in comparison to those with a smaller particle size of ≤0.125 mm. Additionally, a smaller sintering neck and zone between PCP and PES powders are evident (Figure 9a,b and Figure 11a,b). Within the suitable PCP particle size range, laser radiation enables complete PES powder absorption, enhancing liquidity. Effective bonding and encapsulation lead to multiple continuous sintering zones in the parts (Figure 9c,d and Figure 11b). Nonetheless, when employing PCP ≤ 0.105 and ≤0.088 mm, PES powders frequently exhibit inadequate energy absorption, leading to diminished liquidity. Consequently, mechanical strength and sintering quality decrease, as shown in the next section. This leads to larger internal hole size and quantity, along with smaller sintering necks, compared to parts produced using PCP sizes of ≤0.149 and ≤0.125 mm.

Figure 9e–h and Figure 11c,d illustrate that the powders form and interweave into a structural network frame. However, the produced part exhibits many internal pores and low density due to the small size of the PCP particles. The small particle size results in larger surface energy, which easily leads to self-contact. The molten PES promotes the movement of the PCP, causing it to float on the surface of the PES matrix and preventing it from being tightly wrapped. Consequently, the number of internal pores in the PCPC sintered parts (≤0.105 and ≤0.088 mm) is relatively large, leading to lower density and many areas of missing materials on the upper surface. The small particles of PCP hinder the flow of molten PES inside the PCPC part. Although the PES leads the PCP to rearrange, interweaving the PCP and PES into a structural network, the sintered part still has many pores formed inside. In addition, the PES powder does not fully wrap the PCP inside the PCPC sintered parts, so only a few sintered necks are formed. The PCP is unevenly distributed in the PES matrix; this causes some particles to protrude outward, as exhibited in Figure 9g,h. The PCP ≤ 0.125 mm and PES powder are completely compatible, which is because they have different particle sizes; thus, the molten PES can easily flow into the gap between the PCP particles and fill it to produce a PCPC part with excellent quality. Consequently, the SLS parts produced from PCP ≤ 0.125 mm had excellent density, mechanical strengths, and good surface quality compared to others and pure PES SLS parts.

PCPC samples manufactured using different PCP sizes of ≤0.149 and ≤0.125 mm were subjected to SEM analysis prior to and following mechanical testing. SEM images are shown in Figure 11e–h correspondingly. The comparison between the micrographs and deformations of PCPC SLS parts prior to and following mechanical testing (breakage) produced with PCP ≤ 0.149 and ≤0.125 mm is shown in Figure 11e–h. Pre-breakage, the PCPC SLS sample (PCP ≤ 0.125 mm) exhibited superior attributes: prominent sintering neck, minimal pores, and heightened density, as depicted in Figure 11f, compared to the PCPC part formed with PCP ≤ 0.149 mm, shown in Figure 11e. Following the mechanical test that resulted in breakage, the PCPC specimen with a PCP size of ≤0.125 mm showed signs of deformation, particularly at the breakage point, as shown in Figure 11h. On the other hand, the PCPC specimen with a PCP size of ≤0.149 mm displayed a more extensive area of deformation after undergoing the same mechanical test, as depicted in Figure 11g. The findings illustrate that PCPC SLS samples with PCP ≤ 0.125 mm exhibited notable attributes: heightened density, a substantial sintering neck zone, diminished pores, robust mechanical strength, and tightly bonded interfaces, rendering them highly suitable for prospective applications. Consequently, a PCP particle size of ≤0.125 mm is strongly recommended for all forthcoming PCPC composite endeavors, owing to its exceptional qualities.

### 3.4. Effect of PCP Particle Size on Mechanical Properties of PCPC Sintered Parts

**Figure 12:**
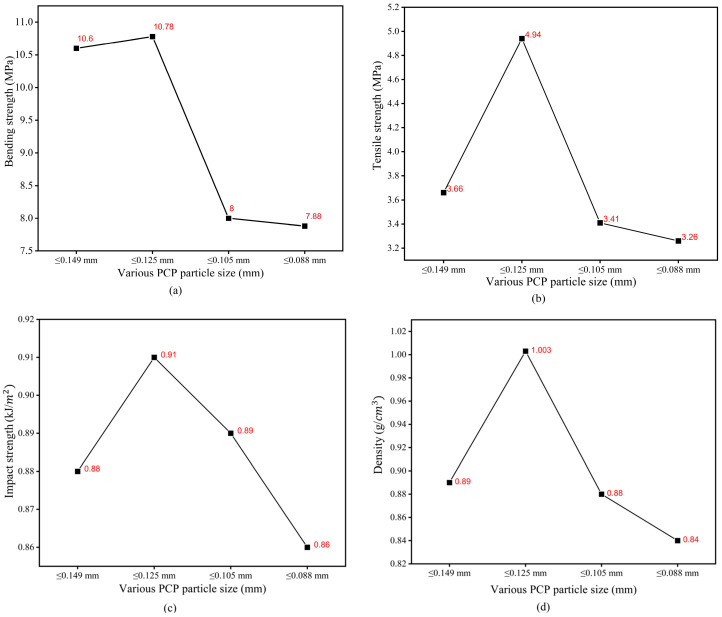
The mechanical strengths (**a**) bending, (**b**) tensile, (**c**) impact strength, and (**d**) density of the PCPC parts.

**Figure 13:**
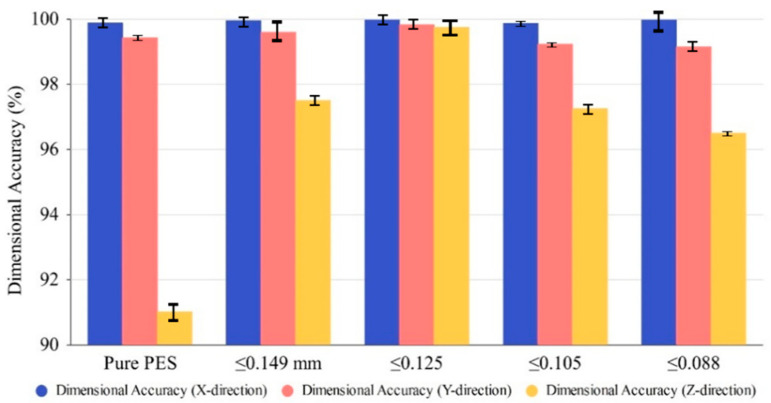
The dimensional accuracies of the PCPC parts in the X, Y, and Z directions with different PCP particle sizes.

The evaluation of mechanical characteristics (density, bending, tensile, and impact) and DA of sintered PCPC parts is crucial for understanding the impact of varying sizes of PCP particles on the functionality and the possible utilization of the resultant PCPC composite. Figure 10, Figure 12 and Figure 13 illustrate variations in DA; surface roughness; density; and impact, tensile, and bending strength profiles of SLS-manufactured components using PCPC with varying sizes of PCP particles. The methods that were utilized to produce PCPC parts were described in a previous section, which are fabricated by ASF 360 SLS. A decrease in the size of PCP particles initially resulted in an enhancement of mechanical properties, encompassing density and impact, tensile, and bending strength, for the PCPC SLS parts. Notably, the highest values recorded were attained at PCP ≤ 0.125 mm, which are 1.003 g/cm^3^ for density, 10.78 for bending strength, 4.94 MPa for tensile strength, and 0.91 kJ/cm^2^ for impact strength. This trend occurred during the transition from PCP ≤ 0.149 mm to ≤0.125 mm. Subsequently, both density and mechanical strengths exhibited a decline. The inclusion of PCP biomass improved the sintering quality of PCPC SLS components, promoting thorough blending and fusion of the PES matrix. This complete fusion and blending fostered strong adhesion among powder particles (PES powder and PCP), subsequently elevating the surface roughness, DA, and mechanical strength of the SLS-manufactured PCPC. During sintering, PES powders frequently experience excessive decomposition, leading to reduced PCPC strength and sintering quality. Nonetheless, the presence of PCP powder mitigated this issue by efficiently absorbing a substantial portion of laser energy.

Comparatively, pure PES sintered components exhibit lower DA than PCPC SLS samples, as demonstrated in Figure 13. This highlights the substantial enhancement in sintering quality of PCPC parts due to PCP, resulting in a uniformly blended matrix powder. This, in turn, fosters strong adhesion between PES powders and PCP, thereby elevating the mechanical strengths of the produced PCPC SLS samples. The decline in mechanical strength and density, as shown in Figure 12, with reduced PCP particle size was linked to weakened PES matrix bonding. This reduction in bonding strength between PES and PCP led to an increase in internal pores between them (Figure 9g,h and Figure 11c,d).

Generally, Figure 12 illustrates the impact of varying PCP particle sizes on the density and mechanical strengths of PCPC SLS components. At PCP ≤ 0.149 mm, the resulting PCPC SLS specimen exhibited a density of 0.89 g/cm^3^, a bending strength of 8 MPa, a tensile strength of 3.41 MPa, and an impact strength of 0.89 kJ/cm^2^. Likewise, diminishing sizes to PCP ≤ 0.125 mm notably boosted mechanical strengths to peak levels for impact, tensile, and bending strength, as well as density. Nevertheless, reducing PCP particles to sizes ≤0.105 and ≤0.088 mm led to notable declines in mechanical strengths of PCPC parts, as displayed in Figure 12, attributed to reduced liquidity and a weaker sintering neck among PCP and PES (Figure 9e,h and Figure 11c,d). As a result, insufficient liquidity causes the cohesiveness between PCP and PES powders to weaken at very small PCP particle sizes. Consequently, with progressive reduction in PCP particle sizes, the mechanical strengths and density of PCPC sintered components consistently reduced, depicted in Figure 12, due to inadequate PES powder melting at small PCP particle sizes, resulting in a relatively weak sintering neck.

Additionally, diminishing PCP particle sizes from ≤0.105 mm to ≤0.088 mm led to decreased sintering connections between molten powders, heightening friction adhesion and surface forces. This resulted in material loss on the upper surface. This phenomenon is clearly shown through 3D morphology parts, as shown in Figure 9g,h. As observed in Figure 9e–h and Figure 11c,d, alongside a notable quantity of internal pores within the sintered parts, a majority of PCP particles appear prominently exposed. These particles lack secure encapsulation within the PES matrix, leading to a less robust bonding interface between the PES matrix and the PCP particles. Primarily, at extremely small PCP particle sizes, molten PES powder forces PCP particles to ascend, causing them to remain on the liquid’s surface. This prevents effective encapsulation by molten PES and hinders the formation of a cohesive solid structure. Consequently, a PCP particle size of ≤0.125 mm was identified as ideal in this investigation, attributed to its superior mechanical characteristics, alongside commendable DA and surface quality, as illustrated in Figure 10, Figure 12 and Figure 13. Hence, a PCP particle size of ≤0.125 mm is optimal for high-quality PCPC composites. 

The mean values underwent statistical analysis, and Table 1 presents repeatability test outcomes, encompassing lower and upper limits along with the standard deviation. The reliability of the tests was established through the observation that all measured strengths were within the 95% confidence interval.

In comparison between the PCPC parts of PCP ≤ 0.125, ≤0.088, and ≤0.105 mm, the PCP ≤ 0.125 mm powder is quite different from PES particles. Therefore, the packing density of the powder bed is increasing; thus, the sintering rate of the particles is optimal under laser beam action, and the melting become sufficient. However, at PCP ≤ 0.088 mm, the PCP particles hinder the melting flow of PES powder, and the molten PES cannot completely fill the gaps between the PCP particles, making the sintered part have many internal pores and low density. However, when comparing the PCPC parts produced at PCP ≤ 0.149 mm and PCP ≤ 0.088 mm, in PCP ≤ 0.149 mm, the molten PES can easily flow into the space between PCP particles and bond them, leading to fewer pores inside the PCPC sintered part and having a higher mechanical strength density and mechanical strength than PCP ≤ 0.088 and ≤0.105 mm, respectively. Subsequent to post-processing, a significant proportion of internal pores were infused with pool wax. This elevation resulted in increased mechanical strength and density: bending strength went from 10.78 to 12.98 MPa, tensile strength from 4.94 to 6 MPa, impact strength from 0.91 to 1.87 kJ/cm^2^, and density from 1.003 to 1.983 g/cm^3^. Furthermore, the PCPC SLS part exhibited an improved surface roughness quality of 4.16 μm after undergoing post-processing, compared to its initial value of 6.87 μm.

### 3.5. Verification of Test Results

**Figure 14:**
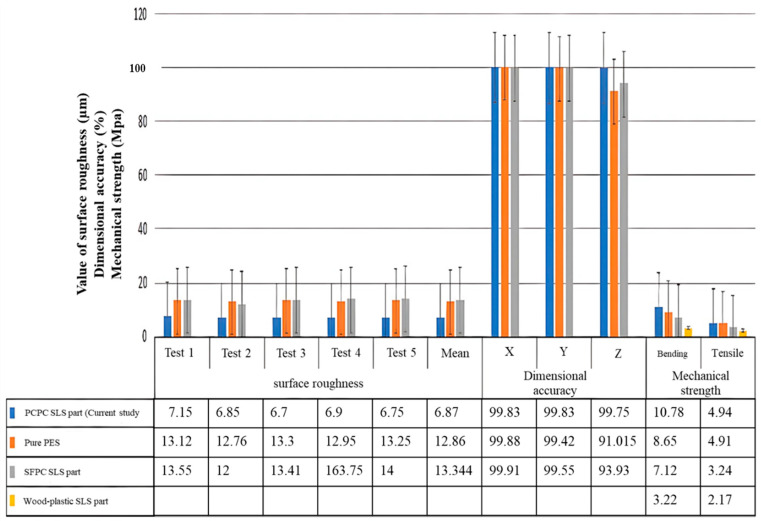
Comparative analysis of mechanical strength, DA, and surface roughness among PCPC, pure PES, and associated biomass composite SLS parts. (Pure PES [32], SFPC SLS part [8], and wood-plastic SLS part [29]).

To ensure the validity of the results, a comparison was conducted between the outcomes obtained and those of pure PES [32], as well as SLS parts made from biomass composites. The comparison was based on factors such as mechanical strength, DA, and surface roughness values. The results of this study were compared to those of other materials, including sisal fiber/PES composite (SFPC) SLS parts [4] and wood–plastic SLS parts [29], as depicted in Figure 14. The results indicate that PCPC SLS parts (PCP ≤ 0.125 mm) exhibit superior roughness, satisfactory DA, and notable mechanical strengths compared to prior findings. The surface roughness of PCPC SLS parts exceeded that of pure PES powder SLS parts, SFPC SLS parts, and wood–plastic composite parts. The PCPC SLS parts (PCP ≤ 0.125 mm) exhibit DA in all three directions in comparison to other combinations. Notably, the Z-direction DA, signifying molding precision of the SLS parts, achieves an impressive 99.75%. Moreover, mechanical strength serves as a pivotal metric for assessing the mechanical behavior and prospective utility of SLS parts. Consequently, parts exhibiting greater mechanical strength deliver superior performance.

**Figure 15:**
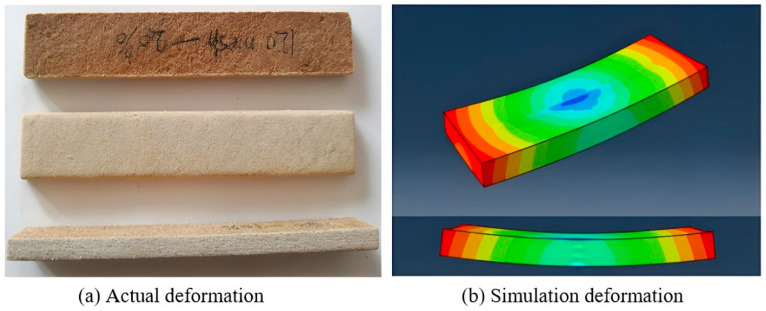
Comparison of the deformations in the actual PCPC parts with those simulated.

In order to guarantee the quality of the PCPC SLS parts, a comparison was made between the deformations of the actual parts and those generated by simulating PCPC powder using the same SLS parameters. These parameters included scanning spacing (0.016 mm), layer thickness (0.14 mm), laser power (12W), preheating temperature (80 °C), and scanning speed (1.8 m/s). Figure 15 displays a comparison between the real and simulated deformations of the PCPC part. As depicted in Figure 15a, the central region of the actual PCPC SLS part did not experience any warping deformation, while the ends of the part exhibited more noticeable warping deformation. In comparison to the simulated part, illustrated in Figure 15b, the actual part demonstrated lesser warpage deformation confirming the high quality and rationality of the produced PCPC part.

## 4. Conclusions

- The main objective of this paper was to identify the optimal particle size of PCP and employ post-processing techniques to fabricate PCPC specimens with favorable mechanical strength, DA, and surface quality through SLS. The findings reveal several significant observations. PCPC SLS parts of superior quality were achieved by using PCP particles with a size of ≤0.125 mm. Initially, PCP particle size led to increased density, surface quality, as well as mechanical strength of the resulting PCPC SLS parts, but beyond a certain threshold, these attributes progressively declined.
- The study also observed that internal voids on the upper surface of PCPC SLS parts were minimized at a particle size of PCP ≤ 0.125 mm, while they increased at particle sizes ≤ 0.088 mm. The uniform distribution of PCPC powders was achieved at a particle size of PCP ≤ 0.125 mm, correlating with the highest recorded values of density (1.003 g/cm^3^), impact strength (0.91 kJ/m^2^), tensile strength (4.94 MPa), and bending strength (10.78 MPa) among the PCPC SLS parts. These attributes exhibited a diminishing trend as the particle size of PCP decreased further to ≤0.105 mm and ≤0.088 mm.
- Additionally, a particle size of PCP ≤ 0.125 mm also demonstrated optimal surface smoothness at 6.87 μm and the most favorable DA in the X, Y, and Z directions, surpassing that of pure PES SLS parts. Post-processing techniques involving wax infiltration significantly improved the mechanical strengths and upper-surface quality of PCPC SLS parts. Surface roughness was reduced to 4.16 μm, density improved to 1.983 g/cm^3^, impact strength increased to 1.87 kJ/cm^2^, tensile strength reached 6 MPa, and bending strength increased to 12.98 MPa in the wax-infiltrated PCPC parts.
- Furthermore, a comparative analysis between the actual sintered PCPC part and its simulated counterpart indicated superior quality in terms of deformation for the actual part, showing lesser warpage deformation than the simulated part.

## Figures and Tables

**Table 1:** Repeatability analysis of mechanical properties for SLS parts manufactured utilizing PCPC with diverse particle sizes of PCP material. The confidence intervals for the means and standard deviation are provided at a 95% level of confidence.

PCPC Parts (10/88 wt/wt)	Bending Strength Test Repeatability			Mean	Confidence Intervals at a 95% Level for the Means	Tensile Strength Test Repeatability			Mean	Confidence Intervals at a 95% Level for the Means	Standard Deviation (SD)	
	Test 1	Test 2	Test 3			Test 1	Test 2	Test 3			Bending	Tensile
Polyethersu-lfone	8.15	8.28	8.23	8.215	Lower = 7.57 Upper = 8.86	4.83	4.9	4.81	4.85	Lower = 4.2 Upper = 5.5	0.065574385	0.047258156
≤0.149 mm	10.9	10.4	10.5	10.6	Lower = 9.95 Upper = 11.25	3.68	3.71	3.59	3.66	Lower = 3.01 Upper = 4.31	0.264575131	0.06244998
≤0.125 mm	10.81	10.8	10.74	10.78	Lower = 10.13 Upper = 11.43	4.9	4.96	4.97	4.94	Lower =4.3 Upper = 5.59	0.037859389	0.037859389
≤0.105 mm	8.2	7.95	7.98	8	Lower = 7.35 Upper = 8.65	3.43	3.39	3.4	3.41	Lower = 2.76 Upper = 4.06	0.136503968	0.02081666
≤0.088 mm	7.91	7.85	7.89	7.88	Lower = 7.23 Upper = 8.53	3.30	3.23	3.25	3.26	Lower = 2.62 Upper = 3.9	0.030550505	0.036055513

## Data Availability

The original contributions presented in the study are included in the article, further inquiries can be directed to the corresponding authors.

## References

[1] Guo Y., Jiang K., Bourell D.L. (2015). Accuracy and mechanical property analysis of LPA12 parts fabricated by laser sintering. Polym. Test..

[2] Danish A., Khurshid K., Mosaberpanah M.A., Ozbakkaloglu T., Salim M.U. (2022). Microstructural characterization, driving mechanisms, and improvement strategies for interlayer bond strength of additive-manufactured cementitious composites: A review. Case Stud. Constr. Mater..

[3] Lupo M., Ajabshir S.Z., Sofia D., Barletta D., Poletto M. (2023). Experimental metrics of the powder layer quality in the selective laser sintering process. Powder Technol..

[4] Li J., Idriss A.I.I., Guo Y., Wang Y., Zhang Z., Zhang H., Elfaki E.A. (2020). Selective laser sintering and post-processing of sisal fiber/poly-(ether sulfone) composite powder. BioResources.

[5] Idriss A.I.B., Li J., Wang Y., Guo Y., Elfaki E.A., Adam S.A. (2020). Selective Laser Sintering (SLS) and Post-Processing of Prosopis Chilensis/Polyethersulfone Composite (PCPC). Materials.

[6] Yu S., Zeng T., Zhao J., Jiang H., Chen Z., Yang Y., Zhong Z., Cheng S. (2023). Preparation and electromagnetic wave absorption properties of PDC–SiC/Si_3_N_4_ composites using selective laser sintering and infiltration technology. J. Mater. Res. Technol..

[7] Lu K., Reynolds W.T. (2008). 3DP process for fine mesh structure printing. Powder Technol..

[8] Qi F., Chen N., Wang Q. (2017). Preparation of PA11/BaTiO3 nanocomposite powders with improved processability, dielectric and piezoelectric properties for use in selective laser sintering. Mater. Des..

[9] Mei S., Wang J., Li Z., Ding B., Li S., Chen X., Zhao W., Zhang Y., Zhang X., Cui Z. (2023). 4D printing of polyamide 1212 based shape memory thermoplastic polyamide elastomers by selective laser sintering. J. Manuf. Process..

[10] Tawfik S.M., Nasr M.N.A., El Gamal H.A. (2019). Finite element modelling for part distortion calculation in selective laser melting. Alex. Eng. J..

[11] Tiwari S.K., Pande S., Bobade S.M., Kumar S. (2018). Assessment of mechanical properties and flammability of magnesium oxide/PA12 composite material for SLS process. Rapid Prototyp. J..

[12] Leach R.K., Bourell D., Carmignato S., Donmez A., Senin N., Dewulf W. (2019). Geometrical metrology for metal additive manufacturing. CIRP Ann..

[13] Gadelmoula A.M., Aldahash S.A. (2019). Effects of Fabrication Parameters on the Properties of Parts Manufactured with Selective Laser Sintering: Application on Cement-Filled PA12. Adv. Mater. Sci. Eng..

[14] Idriss A.I., Li J., Guo Y., Wang Y., Li X., Zhang Z., Elfaki E.A. (2022). Sintering quality and parameters optimization of sisal fiber/PES composite fabricated by selective laser sintering (SLS). J. Thermoplast. Compos. Mater..

[15] Saboori A., Gallo D., Biamino S., Fino P., Lombardi M. (2017). An Overview of Additive Manufacturing of Titanium Components by Directed Energy Deposition: Microstructure and Mechanical Properties. Appl. Sci..

[16] Moshokoa N., Raganya L., Obadele B.A., Machaka R., Makhatha M.E. (2020). Microstructural and mechanical properties of Ti-Mo alloys designed by the cluster plus glue atom model for biomedical application. Int. J. Adv. Manuf. Technol..

[17] Redaelli D.F., Abbate V., Storm F.A., Ronca A., Sorrentino A., De Capitani C., Biffi E., Ambrosio L., Colombo G., Fraschini P. (2020). 3D printing orthopedic scoliosis braces: A test comparing FDM with thermoforming. Int. J. Adv. Manuf. Technol..

[18] Zhang X., Liao Y. (2018). A phase-field model for solid-state selective laser sintering of metallic materials. Powder Technol..

[19] Sofia D., Chirone R., Lettieri P., Barletta D., Poletto M. (2018). Selective laser sintering of ceramic powders with bimodal particle size distribution. Chem. Eng. Res. Des..

[20] Sofia D., Granese M., Barletta D., Poletto M. (2015). Laser Sintering of Unimodal Distributed Glass Powders of Different Size. Procedia Eng..

[21] Yuan S., Bai J., Chua C.K., Wei J., Zhou K. (2016). Material Evaluation and Process Optimization of CNT-Coated Polymer Powders for Selective Laser Sintering. Polymers.

[22] Kim J., Creasy T.S. (2004). Selective laser sintering characteristics of nylon 6/clay-reinforced nanocomposite. Polym. Test..

[23] Sun Z., Yang L., Zhang D., Bian F., Song W. (2019). High-performance biocompatible nano-biocomposite artificial muscles based on a renewable ionic electrolyte made of cellulose dissolved in ionic liquid. Nanotechnology.

[24] Sun Z., Yang L., Zhang D., Song W. (2019). High performance, flexible and renewable nano-biocomposite artificial muscle based on mesoporous cellulose/ionic liquid electrolyte membrane. Sens. Actuators B Chem..

[25] Maringa M., Mwania F., Van der Walt K. (2020). Powder characterization for a new selective laser sintering polypropylene material (Laser PP CP 60) after single print cycle degradation. Int. J. Eng. Res. Technol..

[26] Dowling L., Kennedy J., O’Shaughnessy S., Trimble D. (2020). A review of critical repeatability and reproducibility issues in powder bed fusion. Mater. Des..

[27] Tan J.H., Wong W.L.E., Dalgarno K.W. (2017). An overview of powder granulometry on feedstock and part performance in the selective laser melting process. Addit. Manuf..

[28] Idriss A.I., Li J., Guo Y., Wang Y., Elfaki E.A., Ahmed E.A. (2023). Improved Sintering Quality and Mechanical Properties of Peanut Husk Powder/Polyether Sulfone Composite for Selective Laser Sintering. 3D Print. Addit. Manuf..

[29] Idriss A.I., Li J., Wang Y., Guo Y., Elfaki E.A. (2020). Effects of various processing parameters on the mechanical properties of sisal fiber/PES composites produced via selective laser sintering. BioResources.

[30] Zeng W.L., Guo Y.L. (2010). Research on surface quality enhancement of Wood-Plastic Composite powder SLS parts by post processing. Adv. Mater. Res..

[31] Le D., Nguyen C.H., Pham T.H.N., Nguyen V.T., Pham S.M., Le M.T., Nguyen T.T. (2023). Optimizing 3D Printing Process Parameters for the Tensile Strength of Thermoplastic Polyurethane Plastic. J. Mater. Eng. Perform..

[32] Idriss A.I.B., Li J., Guo Y., Shuhui T., Wang Y., Elfaki E.A., Ahmed G.A. (2021). Selective Laser Sintering Parameter Optimization of Prosopis Chilensis/Polyethersulfone Composite Fabricated by AFS-360 SLS. 3D Print. Addit. Manuf..

[33] (2000). Determination of Charpy Impact Properties—Part 1: Non-Instrumented Test.

